# Low cholesterol? Don't brag yet ... hypocholesterolemia blunts HAART effectiveness: a longitudinal study

**DOI:** 10.1186/1758-2652-13-25

**Published:** 2010-07-13

**Authors:** María Jose Míguez, John E Lewis, Vaughn E Bryant, Rhonda Rosenberg, Ximena Burbano, Joel Fishman, Deshratn Asthana, Rui Duan, Nair Madhavan, Robert M Malow

**Affiliations:** 1 Institute of Neuroimmune Pharmacology, Florida International University College of Medicine, Miami, FL, USA; 2 Department of Health Promotion and Disease Prevention, Robert Stempel College of Public Health and Social Work, and College of Medicine, Florida International University, Miami, FL, USA; 3 Department of Psychiatry and Behavioral Sciences, University of Miami Miller School of Medicine, Miami, FL, USA; 4 Research Division, Zilonis, Inc, Miami, FL, USA; 5 Department of Radiology, University of Miami Miller School of Medicine, Miami, FL, USA

## Abstract

**Background:**

*In vitro *studies suggest that reducing cholesterol inhibits HIV replication. However, this effect may not hold *in vivo*, where other factors, such as cholesterol's immunomodulatory properties, may interact.

**Methods:**

Fasting blood samples were obtained on 165 people living with HIV at baseline and after 24 weeks on highly active antiretroviral therapy (HAART). Participants were classified as hypocholesterolemic (HypoCHL; <150 mg/dl) or non-HypoCHL (>150 mg/dl) and were compared on viro-immune outcomes.

**Results:**

At baseline, participants with HypoCHL (40%) exhibited lower CD4 (197 ± 181 vs. 295 ± 191 cells/mm3, p = 0.02) and CD8 (823 ± 448 vs. 1194 ± 598 cells/mm^3^, p = 0.001) counts and were more likely to have detectable viral loads (OR = 3.5, p = 0.01) than non-HypoCHL controls. After HAART, participants with HypoCHL were twice as likely to experience a virological failure >400 copies (95% CI 1-2.6, p = 0.05) and to exhibit <200 CD4 (95% CI 1.03-2.9, p = 0.04) compared with non-HypoCHL. Low thymic output was related to poorer CD4 cell response in HypoCHL subjects. Analyses suggest a dose-response relationship with every increase of 50 mg/dl in cholesterol related to a parallel rise of 50 CD4 cells.

**Conclusions:**

The study implicates, for the first time, HypoCHL with impaired HAART effectiveness, including limited CD4 repletion by the thymus and suboptimal viral clearance.

## Background

During the course of HIV disease, disturbances of lipid metabolism have been observed long before the introduction of highly active antiretroviral therapy (HAART) and included hypocholesterolemia (HypoCHL; <150 mg/dl) during early stages of the disease and hypertriglyceridemia in late phases [1-6]. However, the relationship between lipids and HIV is complex, dynamic, and bi-directional. For example, recent studies have described different mechanisms by which HIV disrupts cholesterol metabolism [7,8].

Conversely, the virus not only needs cholesterol as a structural component of its membrane, but Gag also attaches to cholesterol, and subsequent HIV-1 particle production requires cholesterol-enriched microdomains or rafts [7,8]. By removing cholesterol, researchers have been able to inhibit *in vitro *HIV-induced syncytium formation, reduce the buoyant density of viral particles, interfere with co-receptor expression, and render cells resistant to infection, thus significantly reducing viral infectivity [7,8]. Accordingly, it has been suggested that lipid-lowering drugs could be used to alter HIV infection [7,8]. However, findings are inconsistent. For example, Claxton and colleagues showed that cholesterol under 160 mg/dl is associated with increased risk of HIV infection [9].

Before reducing cholesterol, numerous factors require consideration, particularly in people living with HIV (PLHIV). For instance, cholesterol and lipid rafts integrity are essential components for the appropriate function of the immune system and the preservation of health [5,10-12]. Authors of this paper and others have shown evidence of some immune dysfunction in PLHIV with HypoCHL [1-4,12].

Nevertheless, these evaluations predated the HAART era, their focus was quite limited, and it is unclear whether these findings are still relevant following the HAART era. Moreover, considering that national guidelines and clinical recommendations for reducing cholesterol are predicated on the idea that "lower is better" understanding the effects of HypoCHL in PLHIV is pivotal [13-15]. What also remains uncertain is how much cholesterol levels can be reduced before adverse outcomes arise. Therefore, to address conflicting results and these uncertainties, the main goal of the present study was to assess *in vivo *the impact of HypoCHL on immune status and viral loads before and after HAART.

## Methods

### Sampling

A gender- and racially-diverse HIV-infected population was screened in January 2005 from the University of Miami Miller School of Medicine clinics affiliated with Jackson Memorial Hospital. HIV-infected participants aged 18 to 55 years were eligible if they were starting HAART regimens. Those who were not naive were eligible if they were without HAART for at least six months.

Patients were excluded if they were non-ambulatory or if they had other conditions that might produce neuropsychiatric or immune/thymus compromise other than HIV (e.g., central nervous system opportunistic infection, head injury with or without loss of consciousness, tumors, major psychiatric disease, developmental disorders, cirrhosis, liver enzymes two standard deviations above normal values, severe malnutrition, or autoimmune diseases). Participants were also excluded if they had a family history of dyslipidemia or were receiving lipid-lowering interventions.

The definition of HAART used for analyses was guided by the published recommendations of the Panel on Antiretroviral Guidelines for Adults and Adolescents of 2008 [16,17].

Those expressing a willingness to participate (98%) and providing written informed consent and a medical release were enrolled and followed up after six months. No significant differences in socio-demographic variables existed between enrolled and non-enrolled participants. The Institutional Review Board at the University of Miami Miller School of Medicine approved the study.

#### Exposure: cholesterol assessment

Fasting blood samples were collected and processed within six hours and sent to a clinical laboratory. None of the PLHIV was acutely ill when blood was drawn. Total cholesterol (TC), high-density lipoprotein (HDL), low-density lipoprotein (LDL), and triglyceride (TG) levels were measured by routine enzymatic methods (KonePro, Konelab). HypoCHL (<150 mg/dL) was defined according to the US National Cholesterol Education Program guidelines [14,15].

#### Immunological and virological outcomes and assessment of HAART efficacy

The main study outcomes are the three laboratory tests and the radiological procedure most related to HAART response: thymus volumes, viral load, and CD4 and naive cells (counts and percentages) [16]. The HIV viral burden was quantified using the Amplicor HIV monitor test (Roche Diagnostic System). The lower threshold for detection at the time of the study was 400 copies/ml. Virological failure was defined using two different thresholds: (1) at least one RNA PCR result greater than 400 copies/ml during treatment follow up; or (2) if baseline burden was >10,000 copies, having had a viral load decline <0.5 log copies/ml [16,17].

The percentage and absolute numbers of T lymphocyte subpopulations CD3^+^/CD4^+ ^and CD3^+^/CD8^+ ^cell counts were determined by flow cytometry as per National Institute of Allergy and Infectious Diseases laboratory protocols. "Immune success" was defined as achieving an increase in circulating CD4^+ ^T cells. However, we also analyzed success by following the World Health Organization (WHO) definition. WHO defines immune success as an increase of 50 CD4 cell counts after six weeks of HAART [18].

The thymus size and naive cells (CD45RA^+^CD62L^+^) were examined as complementary measures of immune reconstitution, which may better reflect HAART response. Flow cytometry was selected because it is frequently used in HIV research, and its analysis is straight forward. On the other hand, TCR Excision Circles (TREC), which is considered the gold standard, is expensive and requires mathematical models for its analyses.

Magnetic resonance imaging (MRI) without contrast was used to determine thymus volume. The MRI was performed using a thoracic surface coil and electrocardiographic gating and consisted of the following sequences: (1) sagittal and coronal pilots; (2) T1-weighted in-phase axial, slice thickness 6 mm, interslice gap 1 mm, four averages; (3) T1-weighted opposed-phase axial, slice thickness 6 mm, interslice gap 1 mm, four averages; and (4) T2-weighted fast spin echo axial fat-suppressed, slice thickness 6 mm, interslice gap 1 mm, four averages.

The thymic volume was calculated using a quantitative method to evaluate the true glandular composition of the thymus, correcting for the degree of fatty infiltration. Quantitative calculation of the thymic volume was performed on T1-weighted in-phase axial slices by multiplying the prevascular mediastinal area on each slice by a correction factor, using fat and muscle signal intensities (SI) to estimate the range of percent glandular composition of the thymus (0-100%):

$$Thymic\;area=Total\;MA\frac{*SI(fat)-SI(M)}{SI(fat)-SI(muscle)}$$

where MA = mediastinum area, SI (fat) = maximal pixel value of mediastinal fat, SI (M) = mean mediastinal signal intensity, SI (muscle) = pectoralis muscle signal intensity. The areas were then summed and multiplied by the 7 mm geometric factor to obtain the thymic volume.

#### Control variables

At baseline and at the six-month follow up, each participant underwent an in-depth assessment, including a detailed interview using standardized research questionnaires covering socio-demographics information, drug and tobacco use habits, HIV infection-related data (i.e., stage of HIV infection), and complete past and present medical and medications history. Medication use history included previous exposure to antiretrovirals, lipid-lowering medications, and viral hepatitis treatment. These questionnaires have been used in our previous studies [19,20]. Adherence was calculated using both pharmacy records and a standardized antiretroviral adherence questionnaire. After visit procedures were completed, a medical chart and pharmacy records were abstracted and patient information was validated.

Alcohol consumption assessments included widely-used standardized and validated brief screening questionnaires: The World Health Organization's Alcohol Use Disorders Identification Test (AUDIT) has three questions on alcohol consumption, three questions on drinking behavior and dependence, and four questions on the consequences or problems related to drinking. The ADS (Alcohol Dependence Scale) is a widely used research and clinical tool that provides a quantitative measure of the severity of alcohol dependence. Based on criteria established by the National Institute of Alcohol Abuse and Alcoholism and American Association guidelines, men who reported >14 and women >7 drinks/week were enrolled in Group 1 (hazardous drinkers), while those who reported fewer drinks were included in Group 2 (non-hazardous drinkers).

Nutritional measures included 24-hour dietary intake, anthropometrics, and albumin levels. Body weight and height were measured and used to calculate body mass index (BMI; weight in kilograms divided by height in meters squared).

### Statistical analyses

Data were analyzed using SPSS 15.0 (SPSS, Inc., Chicago, IL, USA), and p values <0.05 were considered significant. Both cross-sectional and longitudinal analyses were used. Following descriptive statistical analyses, mean variables were compared using Student's t-test and one-way analysis of variance (ANOVA) procedures to determine the covariates for inclusion in the univariate analyses and in the multivariate model (e.g., age, race/ethnicity, education level, stress, depression). Fisher's exact tests were used when appropriate. Correlations among the main variables of interest were examined with Pearson's coefficients. Chi-square, Student's t-test, ANOVA and analyses of covariance (ANCOVA) were used to evaluate differences in lipid levels, HIV viral load, and immune parameters of interest (thymus, lymphocyte phenotype, and naive cells) between cases and controls at baseline and after six months of receiving HAART.

These tests were also used to compare older and younger participants (<45 years old and ≥45 years old). It should be noted that the lower age limit in the literature for "older" is 45 years of age, and since HIV accelerates the process of aging, we decided to use this cut-off point.

Changes in lipid profiles between the baseline and the six-month evaluations were assessed using Student's t-test for paired samples. In addition, ANCOVA were used to test if, after six months on HAART, viral load and CD4 changes were different between the HypoCHL and the non-HypoCHL groups after controlling for age, gender, adherence, and their baseline status. Viral load was transformed to the log10 scale for modeling. An indicator variable for a CD4 count of less than 200 cells/mm^3 ^was created for use in modeling.

Univariate analyses were used to calculate odds ratios (OR) and 95% confidence intervals (CI). Finally, outcomes and observed covariates that were significantly associated with immune status in univariate analysis were then utilized as covariates in a multivariate model. In addition, potential participant predictors (i.e., gender, race/ethnicity, CDC disease status, drug use, and BMI) were selected based on their importance in the HIV literature and were added to the model. Non-significant variables (p = 0.05) were removed, beginning with the least significant variables, until the final full model was determined. Model statistics included adjusted OR, 95% CI, and their corresponding p values. For the final model, efficacy was estimated according to the principle of intention to treat, considering all missing values as failures.

## Results

### Study population characteristics

For analyses, the sample included 165 participants who did not receive cholesterol-lowering medication and who had baseline and six-month follow-up blood samples drawn. The mean TC level of the sample was 173 ± 43 mg/dl (52-324 mg/dl) with HypoCHL present in 40% of participants at baseline and 33% at week 24. Only 5% had more than 250 mg/dl of TC. TC levels were unrelated to TG levels and to malnutrition (see Table 1).

**Table 1:** Baseline sociodemographic information for HIV-infected participants with and without hypocholesterolemia

Variables	Total cholesterol <150 mg/dL (n = 68)	Total cholesterol >150 mg/dL (n = 97)
**Age in years**	39.8 ± 7.9	41.1 ± 7.2
**Men**	72%	68%
**Women**	28%	32%
**Black**	65%	55%
**Hispanic**	33%	29%
**White**	0%	16%
**Annual income**		
**Less than $10,000**	89%	88%
**$11,000-$20,000**	9%	10%
**More than $20,000**	2%	2%
**Cigarette smoker**	81%	80%
**Hazardous alcohol use**	64%	48%
**Drug use**	42%	39%
**Albumin in mg/dl**	4.0 ± 0.4	4.1 ± 0.5
**Body mass index in kg/m**^**2**^	26.2 ± 6.2	27.4 ± 6.7
**Number of years diagnosed with HIV**	9.7 ± 6.6	8.7 ± 5.6

Note: Values are mean ± standard deviation or percentages. The data represent descriptive statistics and comparisons of key variables between individuals with and without hypocholesterolemia. No significant differences in socio-demographic characteristics were found between groups.

Table 1 displays the demographic and clinical characteristics of the sample by cholesterol group. Groups were comparable on sociodemographic variables. However, patients with HypoCHL were less likely to be white (95% CI 0.0-0.48, p = 0.001) and more likely to be hazardous alcohol users. Since we have previously shown that liquor/spirits (e.g., rum, whisky, vodka) has more severe adverse effects than other types of alcohol (beer or wine), we explored the potential effect of specific alcoholic beverages while controlling for age. HypoCHL individuals were more likely to be hazardous liquor users than non-HypoCHL youths (OR = 2.2, 95% CI 1-5.3, p = 0.05).

HIV medication use, including past and current prescription regimens, and the proportion of protease inhibitor- versus non-nucleoside reverse transcriptase inhibitor-containing regimens was not significantly different. The difference between the two groups in adjusted adherence to medication was only 3%.

Important for understanding the relationship between HypoCHL and immune response is whether the results are moderated by overall health indices (i.e., albumin and anthropometrics). As shown in Table 1, albumin and BMI were comparable between groups. Also to be considered was the effect of viral hepatitis, but the exclusion of participants with cirrhosis or liver enzymes two standard deviations above normal values highly reduced the likelihood that patients with active hepatitis C would be enrolled and affect the outcomes. In addition, the JMH clinic that served as the recruitment site conducts an annual screening for viral hepatitis among PLHIV clients. Once positive, clients are immediately referred to treatment and our treatment records had no evidence of such events.

#### Baseline cholesterol and immune parameters

Immune results are displayed in Table 2 and depict men and women in the mid-range of illness (CD4 numbers between 150 and 500). Overall, the analyses revealed that relative to the control group, HypoCHL subjects had significantly less circulating total T cells and fewer CD4 (197 ± 181 vs. 295 ± 191 cells/mm^3^, p = 0.02) and CD8 counts. Nonetheless, 25% of PLHIV with more than 500 CD4 cell counts exhibited HypoCHL, indicating that it was not restricted to advanced stages of the disease.

**Table 2:** Baseline immune measures in HIV-infected participants with and without hypocholesterolemia

Variables	Total cholesterol <150 mg/dL (n = 68)	Total cholesterol >150 mg/dL (n = 97)	p value
**Total T lymphocytes cells/mm**^**3**^	1234 ± 737	1505 ± 650	0.02

**CD4 cells/mm**^**3**^	197 ± 181	295 ± 191	0.02

**CD8 cells/mm**^**3**^	913.1 ± 390	1146.5 ± 580	0.001

**Thymus volume cc**^**3**^	8.5 ± 4.6	9.8 ± 2.0	0.4

**Viral load HIV copies/mL**	208,614 ± 42,369	150,441 ± 27,567	0.2

Note: Values are mean ± standard deviation. The p values represent t-tests comparing variables between individuals with and without hypocholesterolemia. Groups significantly differed in all lymphocyte measurements.

Univariate analyses demonstrated that members of the HypoCHL group were twice as likely to have CD4 counts below 200 cells as those in the control group (95% CI 1.0-4.13, p = 0.03). Analyses of immunological data in older PLHIV revealed a significant relationship between HypoCHL and low absolute CD8+ T cell counts (913.1 ± 390 vs. 1146.5 ± 580 cells/mm^3^, p = 0.003). Older participants with HypoCHL also had significantly lower percentages of both naive CD8+ (25 ± 8 vs. 75 ± 15) and naive CD4+ cells (13.0 ± 2 vs. 26.5) compared with the older non-HypoCHL subgroup. Relative to the young group with normal values, the younger group with HypoCHL had significantly lower CD8+ T cell counts (826.7 ± 50 vs. 1175.5 ± 74, p = 0.002), with a tendency for lower CD4 absolute numbers (204.6 ± 29 vs. 268.6 ± 23.5, p = 0.06) and CD4 (p = 0.08) naive cells.

#### Baseline cholesterol and HIV viral loads

TC and viral load levels were significantly correlated (r = 0.3, p = 0.02). Although not statistically significant, subjects with HypoCHL did not exhibit lower, but rather higher viral loads (see Table 2). Moreover, in contrast to results from *in vitro *studies, univariate analyses indicated that participants with non-HypoCHL were more likely to have an undetectable viral load than those with HypoCHL (OR = 3.5, 95% CI 1.2-11, p = 0.01).

#### Longitudinal analyses of viral load response after 24 weeksof HAART

An undetectable viral load was achieved by 69 participants (42%). Sixty-six subjects (40%) had a viral load decline >0.5 log copies/ml, but still detectable viral load at the last visit, while the remaining participants had no decline or declined ≤0.5 log copies/ml. It is noteworthy that despite similar medication adherence, a significantly greater proportion of HypoCHL participants had a virologic failure compared with the control group (19% vs. 9%, p = 0.04), and they had a non-significant reduction of 32,070 HIV copies (-0.6 log). In contrast, in the non-HypoCHL group, a significant decrease in viral load after 24 weeks (87,440 HIV copies = 1.6 log, p = 0.004) was observed.

**Figure 1:**
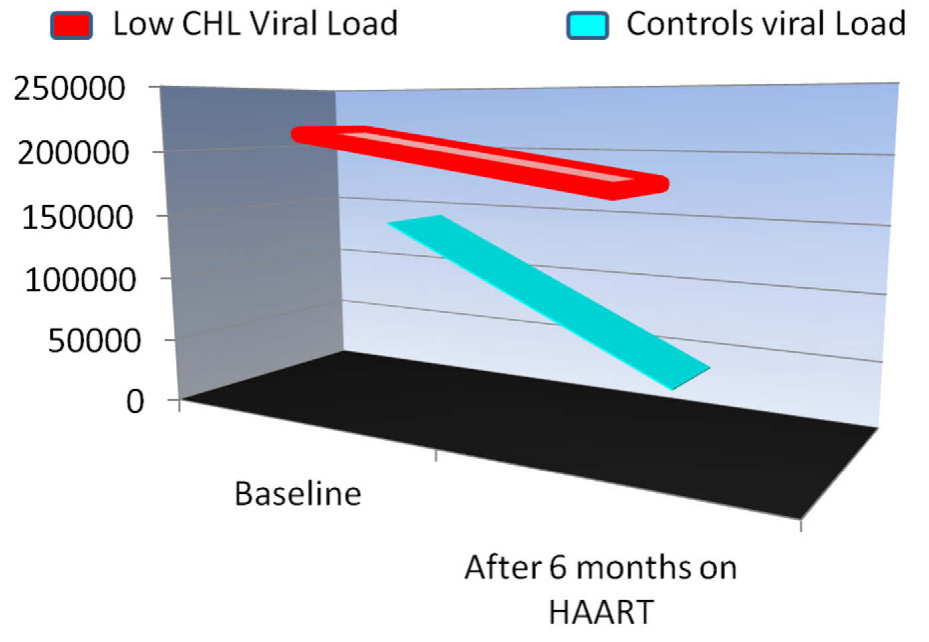
**Viral loads before and after HAART by cholesterol groups**. Figure 1 indicates trends in viral loads between HIV positives with Hypocholesterolemia (n = 68) and controls (n = 97), starting at the visit before HAART, and extending over a six-month period, after adjustment for adherence. As depicted in the figure, a significant drop in viral load was attained in the individuals with cholesterol values above 150 mg/dl.

After controlling for age, gender, and the baseline log viral load, an ANCOVA model showed that the six-month log viral load of the HypoCHL group was significantly higher (p = 0.04) by 0.93log, than the non-HypoCHL group. Figure 1 illustrates the significantly different slope of change between the two groups using the actual values. Analysis showed a strong negative correlation between CD8 cell counts and viral load at week 24 (r = 0.38, p = 0.006). After controlling for adherence, participants without HypoCHL experienced a greater drop (p = 0.012) in mean viral loads with HAART than did participants with HypoCHL (see Figure 1).

#### Longitudinal analyses of immune recovery after 24 weeks of HAART

**Figure 2:**
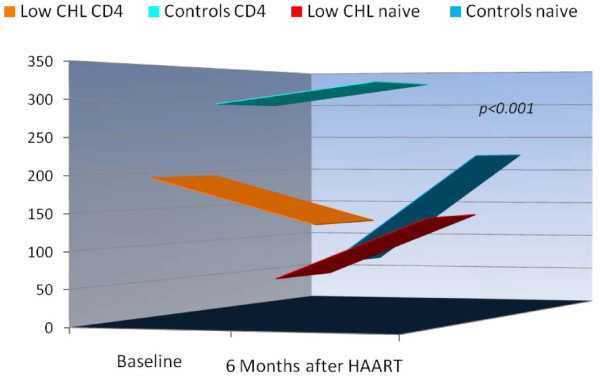
**T cell replenishment with HAART by cholesterol groups**. Data represent mean changes in CD4 and total naive T cell counts after HAART in the two groups and were tested with paired-sample t-tests. Both CD4 and total naive subsets controls (n = 97, in the blue colors) exhibited a significant improvements after HAART (p = 0.001). On the contrary, PLHIV with HypoCHL (n = 68, orange and red), showed a reduction in total number of CD4 cells, and only a non-significant increase in total naive cells (CD3+CD45RA+CD62L+).

After six months on HAART, PLHIV with persistent HypoCHL exhibited significantly lower thymus volumes (6.9 ± 4.4 vs. 10.8 ± 7 cc^3^, p = 0.03). Figure 2 shows that the therapy induced a limited increase of CD4+ T cells in the HypoCHL group compared with controls (+18 vs. +102 cells/mm^3^, p = 0.001). Analysis of naive cells was more alarming, as participants in the control group showed increases in CD4+ T cells (+29 cell counts/mm^3^), compared with values obtained from HypoCHL individuals who exhibited a decline (-59 cells/mm^3^). These changes parallel those of thymus volumes, while controls exhibited an increased in thymus volumes (+2.4 cc3, p = 0.09), those with HypoCHL had a significant decrease (-2.8 cc3, p = 0.04).

Accordingly, participants with HypoCHL were twice as likely to have <200 CD4 cell counts (OR = 1.6, 95% CI 1.03-2.88, p = 0.04) and five times less likely to achieve more than 500 CD4 cell at six months (OR = 5.4, 95% CI 3.1-9.4, p = 0.0001) than controls. Both groups exhibited a fall in total number of CD8^+ ^T lymphocytes. However, decreases were more evident in the HypoCHL group. As a result, after six months of therapy, the HypoCHL group exhibited significantly lower total CD8 cell counts (823 ± 448 vs. 1194 ± 598 cells/mm^3^, p = 0.001). Additional analyses revealed that HAART-induced expansion of the naive subset was better in participants with normal baseline cholesterol (Figure 2).

Given the observed deleterious effects of HypoCHL, we then examined if these effects had a threshold on immune responses. To address this question, the HypoCHL group was dichotomized into those with baseline cholesterol values above (n = 40) and below the group mean (120 mg/dl, n = 28). The outcome variable was viro-immune measures at the last visit. Groups significantly differed in viral loads, total T cell, and CD8 counts; those with less than 120 mg/dl exhibited the worst viro-immune profile (CD3 = 1011.9 ± 110 vs. 1427 ± 109 cells/mm3, p = 0.03; CD8 = 694.5 ± 89 vs. 1110 ± 103 cells/mm^3^, p = 0.01; and log viral load 2.9 ± 2.2 vs. 4.7 ± 1.2 copies, p = 0.03).

### Final analyses of HAART efficacy

After adjusting for age, gender, CDC status, and other covariates (e.g., BMI), HypoCHL was the only independent risk factor of virological failure after six months of HAART (RR = 1.7, 95% CI 1-2.6, p = 0.05). In fully adjusted models (sociodemographics, baseline CDC stage, drug use, and adherence), HypoCHL (RR = 7.7, p = 0.002), thymus volume (above or below 9, which is the mean of the study population, RR = 0.07, p = 0.03), hazardous liquor use (RR = 1.57, p = 0.05), and detectable viral loads at the last visit (RR = 8.6, p = 0.04), significantly predicted CD4 below 200 cell counts (see Table 3).

**Table 3:** Multivariate analyses for CD4 below 200 cell counts

**Predictors of CD4 below 200 cell counts/mm**^**3**^			
MODEL	MULTIVARIATE RR	95% CI	p Value
Hypocholesterolemia	7.7	2.1-27	0.002
Small thymus	0.07	0.06-0.8	0.03
A persistent viral load	8.6	1.0-70	0.045
Hazardous liquor use	1.6	1.0-2.3	0.053

RR represents the final Logistic regression model predicting CD4 cell counts below 200 cells/mm^3 ^after 6 months of HAART. Significance was set at 0.05. Model statistics computed include adjusted OR, 95% CI, and their corresponding p values. Hypocholesterolemia, thymus, and viral load were set as dichotomous variables.

**Figure 3:**
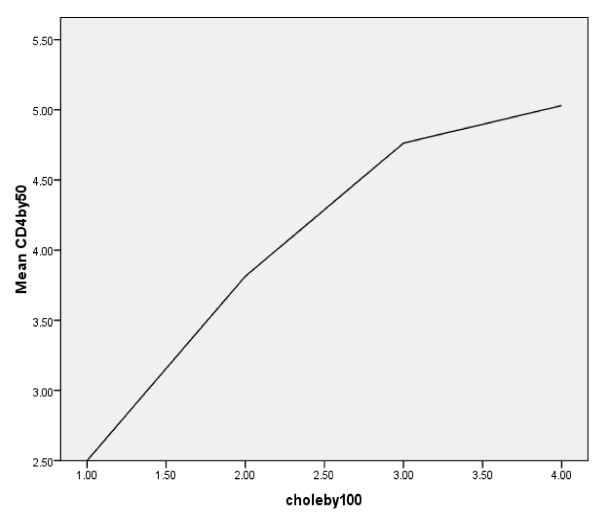
**CD4 counts by cholesterol**. Mean CD4 cell counts of the total group were stratified by increases of 50 cell counts and plotted against cholesterol groups in increments of 100 mg/dl. R and p values were calculated by linear regression analysis. As depicted, CD4 cell counts increased linearly with increases in cholesterol.

To examine a possible dose-response relationship, cholesterol was converted into an ordinal variable with four levels (100-150, 151-200, 201-250, and >251 mg/dl) and mean CD4 counts were compared. As depicted in Figure 3, the results suggested a dose-response relationship between TC and CD4 cell counts after HAART (p < 0.05). Based on this analysis, every increase of 50 mg/dl in TC was accompanied by an increase in CD4 of 50 cell counts.

## Discussion

Although primarily high TC has been publicized as unhealthy [21,22], results from this longitudinal study indicate that low cholesterol in PLHIV may be as insalubrious or worse than high cholesterol. In addition, our findings highlight the importance of cholesterol in increasing immune resilience and maintaining thymus function in older PLHIV.

Equally important, HypoCHL was fulfilling all the established IARC (International Agency for Research on Cancer) criteria to attribute morbidity to a given exposure [23,24]. These are:

a. Our results were *consistent with previous studies and highly significant *(RR = 7.7, p = 0.002).

b. *The data indicated a temporal relationship *between cholesterol status and immune responses.

c. Analyses suggest *a dose-response relationship*.

d. Our findings demonstrate, for the first time, *a mechanism mediating the deleterious effect of HypoCHL *on T lymphocytes and, thus, on HAART effectiveness.

e. *Reversibility of effects*: Participants who normalized their cholesterol values did not exhibit the deleterious profile of those who demonstrated persistent HypoCHL.

Several biological factors may be considered to account for an inverse relationship between cholesterol levels and immune impairment. The deleterious effects of HypoCHL on the thymus may explain our findings of fewer circulating total T, CD4, and CD8 cells in these subjects, but also Rodriguez *et al *[25] observed that statins blunt short-term CD4 gains with HAART. In non-HIV infected participants, Muldoon and colleagues [26] also observed a depletion of CD4 and less interleukin-2 (IL-2) release in response to mitogen stimulation in HypoCHL participants.

Considering that IL-2 has been shown to prevent T cell apoptosis, one can postulate that cholesterol's effects on IL-2 and the thymus may explain the fewer cells observed with HypoCHL in our participants [27,28]. Moreover, last year, an animal study demonstrated that cholesterol modifications can affect IL-7 production, which is necessary for thymus expansion and production of naive cells [29].

Therefore, one can postulate that HypoCHL, by affecting IL-2 and IL-7, may decrease T cell production, proliferation, survival, and repletion under HAART. Given the thymus's sensitivity to metabolic changes, and the role of cholesterol in lipid rafts, and considering that proliferation and generation of new cells is a cholesterol-demanding process, our findings were not wholly unexpected. Nevertheless, the exact mechanism(s) mediating HypoCHL's effects on thymus size and function requires further investigation.

Recently, *in vitro *studies have revolutionized our understanding of HIV infection by demonstrating that HIV is dependent on cholesterol [30-34]. The virus not only needs cholesterol as a structural component of its membrane, but it also takes advantage of membrane lipid rafts for the attachment and entry into the cells [30-34]. Using cell lipid rafts as a point of entry allows the virus to exploit the high concentration of cellular receptors CD4, CCXCR4, and CCR5 that are required for virus entry [5,31,32]. Nonetheless, it has been argued that cholesterol modulates HIV entry independently of its ability to promote lipid raft formation [33].

Finally, Nef-enhanced cholesterol synthesis is thought to sustain virion assembly in lipid rafts and budding from the membrane. By removing cholesterol, researchers have been able to inhibit *in vitro *HIV-induced syncytium formation and reduce the buoyant density of viral particles and virus infectivity [35]. This has led to advocacy of cholesterol-lowering interventions, as a beneficial strategy for both preventing and treating HIV [29-33,36]. Currently, the field is confronted with contradictory and often discouraging data in human subjects [25,37-40]. Three studies found no beneficial effects, while two other studies demonstrated that cholesterol-lowering drugs increased viral replication, particularly in individuals with low cellular cholesterol [25,37-39,41].

Our study results showed that participants with HypoCHL exhibited higher, rather than lower, viral loads. The discrepancies between our results and those from *in vitro *experiments may be related to several factors. First, *in vitro *research focuses on cell membrane cholesterol, and we only evaluated plasma levels [29-34,42]. Second, in laboratory experiments, only cells that over-express HIV co-receptors of entry have been used. Third, those researchers utilizing experimental models cannot examine the systemic effect of such a manipulation.

Our findings are probably associated with a weaker immune response toward the virus, as indicated by the significant correlations between CD8 counts and cholesterol on the one hand and the negative correlation between CD8 and viral loads on the other. CD8 anti-HIV activity is sufficiently well- documented, so that such further discussion may be unnecessary [43]. It is also possible that during the acute phase of infection (*in vitro *model), HypoCHL may help to contain viral replication and then contribute to immune suppression and poor viremic control once an individual enters the chronic stage of infection.

Finally, both HIV and alcohol abuse have been associated with altered gut permeability and microbial translocation, which are linked with immune activation, leading to increase viral replication [44,45].

Few studies have focused on CD8^+ ^T cells and even less have evaluated naive CD8 cell changes. Only one other study, in non-HIV-infected individuals, has reported the effect of age on naive CD8 T-cell counts [46]. Our results extend the prior publication by demonstrating that the depletion of CD8 naive cells is significantly associated with HypoCHL. Considering that CD8 lymphocytes, particularly naive cells, are involved in the body's response to novel class antigens (i.e., viruses and malignant cells), our findings offer a mechanism for explaining why respiratory infections and cancer may be important causes of morbidity and mortality in the HypoCHL aging population.

In summary, this study indicates that HypoCHL is significantly associated with impaired viro-immune response in participants with and without HAART. Our findings have important clinical implications. First, our results highlight the value of further elucidating the impact of TC and lipid-lowering drugs on the immune systems of PLHIV because any additional suppression may produce harm. Second, it emphasizes the need to further evaluate clinical guidelines regarding TC levels in PLHIV. It also calls for caution in advocating for lipid-lowering medications as a preventive or adjuvant therapy against HIV.

In the HAART era, when physicians are particularly concerned with hypercholesterolemia increasing the risks of viral infectivity and cardiovascular disease, our data indicate that HypoCHL is as clinically relevant. However, some limitations of our study should be noted. First, the study only indicates an association and cannot establish a causal relationship. However, several factors reduce the likelihood of our findings being due to chance: the magnitude of the association between HypoCHL and HAART-blunted responses; the biological plausibility of this association; the temporality of the relationship; the dose-response relationship; and the longitudinal design of the study.

Additionally, it is important to note that the size of the current study allows only moderate estimates of change in the lipid profiles following HAART. Also, our results are limited to a single cohort, followed in a clinical setting where antiretrovirals are readily available, compliance is high, and black patients are over-represented. Whether the results will generalize to different populations needs future clarification.

Even though our data reflect only the initial six months of therapy, previous studies indicate that most HAART-related changes occur within this time period [22]. Therefore, larger samples with longer follow-up periods are recommended to corroborate our findings. Finally, we did not include intracellular measurements of cholesterol since, in practice, clinicians measure blood levels, not intracellular cholesterol, rendering our results of greater applicability to clinical settings.

## Conclusions

Despite these limitations, our *in vivo *study of how HypoCHL affects PLHIV extends the literature in several ways. First, to our knowledge, this is the first cohort study of lipids in antiretroviral-treated patients that considers thymus volumes and measurements of recent T cell outputs in addition to CD4 cell counts and viral load. Second, the study design permitted us to examine the importance of HypoCHL in HIV disease at baseline and the biological effects of HypoCHL on HAART effectiveness.

Finally, we were able to avoid the confounding effects of individuals in therapy at diverse times with all participants initiating treatment at the same point. Currently, comprehensive studies to elucidate the role of cholesterol in health and disease are needed to help inform public health authorities and health care providers on treatment decisions.

## Abbreviations

HAART: highly active antiretroviral therapy; HypoCHL: hypocholesterolemia; Non-hypocholesterolemia: Non-HypoCHL; TC: total cholesterol; HDL: high-density lipoprotein; LDL: low-density lipoprotein; TG: triglyceride; PLHIV: people living with HIV; BMI: Body mass index.

## Competing interests

The authors declare that they have no competing interests.

## Authors' contributions

MJM conceived the study, participated in its design and coordination, and drafting of the manuscript. JF interpreted the thymus MRIs. DA and NM carried out the immunological studies. RMM, VEB, and RR participated in the drafting of the manuscript. JEL and RD participated in the statistical analysis and drafting of the manuscript. XB participated in the formatting of the manuscript and prepared the references' used for the article. All authors read and approved the final manuscript.

## Authors' information

Results from this study were presented previously in part in:

Míguez MJ, Lewis JE and Malow R. Immune System Connection to Low Cholesterol in the HAART Era. The United States Conference on AIDS (USCA), Sept 18-21, 2008, Fort Lauderdale, Fl.
